# Targeted treatment of hypophosphatemia with trametinib in HRAS-related mosaic RASopathy

**DOI:** 10.1186/s13023-025-03801-5

**Published:** 2025-07-03

**Authors:** Adil Mirza, Corinne Rossi, Andreas Kulozik, Rouzbeh Banan, Felix Sahm, Semi Harrabi, Steffen Syrbe, Daniela Choukair

**Affiliations:** 1 Medical Faculty of Heidelberg, Department of Pediatric Oncology, Hematology and Immunology, Heidelberg University, Hopp- Children’s Cancer Research Center (KiTZ), Heidelberg, Germany; 2 Medical Faculty of Heidelberg, Department of Neuropathology, Institute of Pathology, and Clinical Cooperation Unit Neuropathology, Heidelberg University, DKFZ, Heidelberg, Germany; 3 Medical Faculty of Heidelberg, Department of Radiotherapy, Heidelberg University, University Hospital Heidelberg, Heidelberg, Germany; 4 Medical Faculty of Heidelberg, Division of Pediatric Epileptology, Centre for Pediatrics and Adolescent Medicine, Heidelberg University, Clinic 1 Im Neuenheimer Feld 430, 69120 Heidelberg, Germany; 5 Medical Faculty of Heidelberg, Division of Pediatric Endocrinology and Diabetes, Centre for Pediatrics and Adolescent Medicine, Heidelberg University, Clinic 1, Heidelberg, Germany

## Abstract

Schimmelpenning-Feuerstein-Mims syndrome (SFMS) is a rare mosaic RASopathy associated with epidermal nevi, neurological abnormalities, and increased cancer risk. We report a 2-year-old girl with *HRAS*-related SFMS, aggressive orbital rhabdomyosarcoma (eRMS) and severe hypophosphatemic rickets resistant to standard therapies. Treatment with the MEK inhibitor trametinib improved phosphate regulation, reducing FGF23 levels, and led to rapid developmental progress, including independent walking. After 29 months, the patient remains in cancer remission with stable phosphate levels. This case highlights trametinib’s potential in managing complex manifestations in SFMS and suggests MEK inhibitors as promising for treating mosaic RASopathies.

## Abstract (article summary)

A 2-year-old with HRAS-related Mosaic RASopathy complicated by rhabdomyosarcoma, and hypophosphatemia showed marked improvement with MEK inhibitor trametinib, highlighting its potential as targeted treatment.

## Introduction

In 1957, Schimmelpenning initially documented the presence of an epidermal nevus along with neurological anomalies [1]. In 1962, Feuerstein and Mims reported a case involving a linear nevus, epilepsy, and intellectual disability [2]. Schimmelpenning-Feuerstein-Mims syndrome (SFMS, OMIM#163200) has been characterized as the triad of sebaceous nevus, seizures, and intellectual disability, with variable cooccurrences of these symptoms together with extracutaneous manifestations that can exhibit variability among cases, encompassing central nervous system, ocular, skeletal, cardiovascular and urologic abnormalities [3- 7].

SFMS is a mosaic RASopathy caused by postzygotic somatic activating variants in the proto-oncogenes *HRAS*, *KRAS*, and *NRAS* that act as small GTPases in the RAS/mitogen-activated protein kinase (MAPK) pathway, which promote variable organ manifestations and proliferative potential [8- 12].

Somatic variations occur during embryonic development [13]. The timing and location of de novo variation are responsible for the variable cutaneous and extracutaneous manifestations [8].

Beyond the characteristic features, variants in the RAS oncogenes also correlate with a predisposition to certain cancers, notably embryonal Rhabdomyosarcomas (eRMS) [14, 15, 16]. Additionally, rare manifestations, such as hypophosphatemic rickets, acknowledged as cutaneous skeletal hypophosphatemia (CSHS), have been reported [17].

Novel targeted inhibitors of the RAS/MAPK pathway are promising treatments for different manifestations of the spectrum of RASopathies [10, 18, 19, 20, 21]. Here, we present a case of a 2-year-old girl with mosaic RASopathy complicated by eRMS and CSHS who was treated with trametinib as a systemic MEK inhibitor.

### Case report

**Fig. 1:**
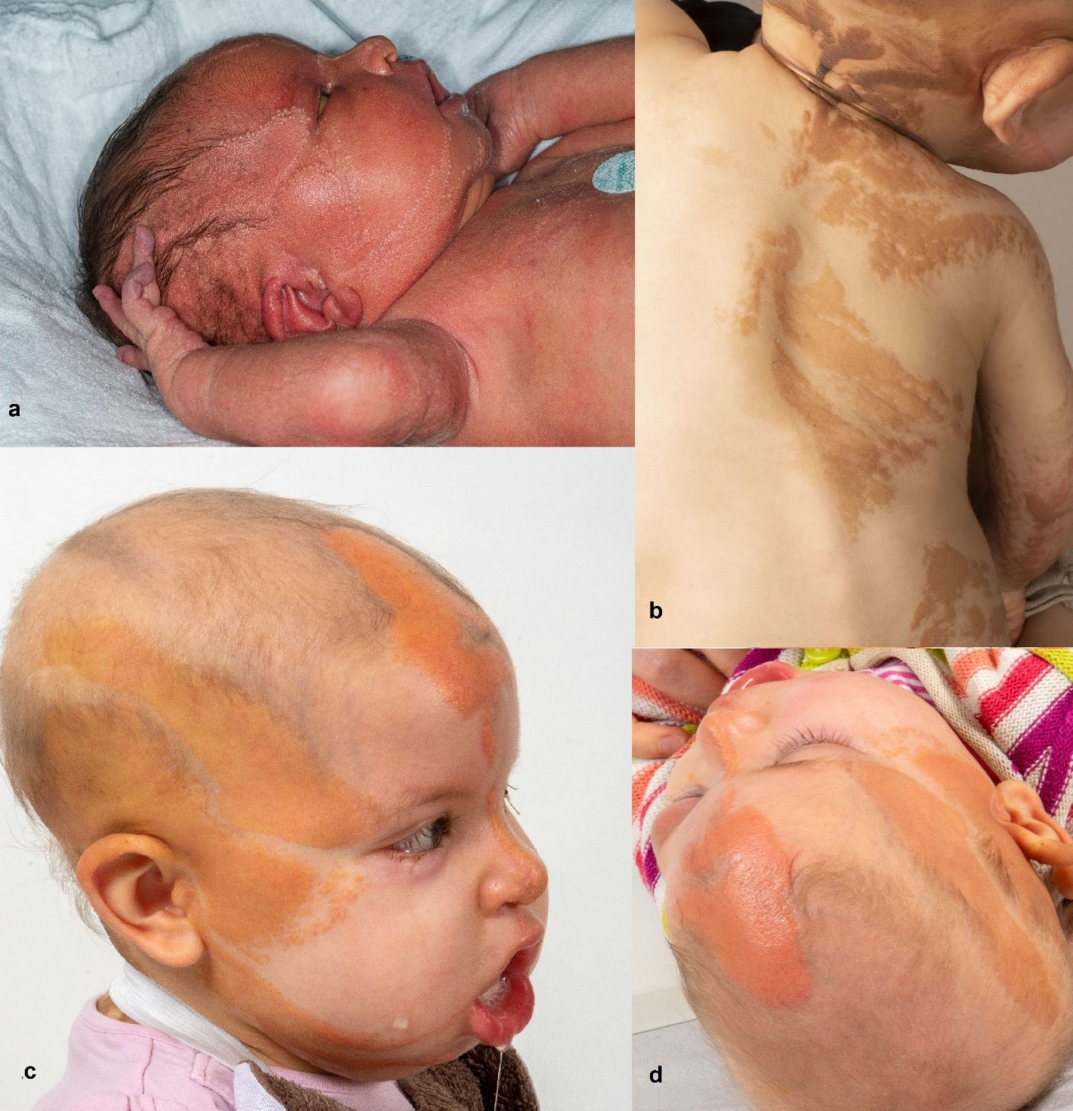
Clinical presentation at 2 days of life (**a**), 12 (**b**) and 20 months of age (**c**, **d**). Nevi and skin changes were more prominent on the right side, reflecting mosaic status of HRAS-variation. Skin texture and color changes together with growth of hair improved during treatment

A eutrophic premature infant born at 36 weeks following premature rupture of membranes presented distinctive clinical features, including hypopigmented, erythematous, hairless plaques along the Blaschko lines, predominantly on the right side of the head, trunk, and extremities (Fig. 1). Further examination revealed hymenal prolapse and bilateral convergent strabismus due to symmetrical abducens paresis. The girl experienced respiratory distress, necessitating continuous positive airway pressure support for the initial 48 h and feeding difficulties from congenital cranial dysinnervation affecting multiple cranial nerves. As a result, feeding progression was delayed, and the infant was discharged with a gastric tube. No seizures were reported, and cranial magnetic resonance imaging showed no abnormalities. A clinical diagnosis of Schimmelpenning-Feuerstein-Mims syndrome was confirmed through genetic analysis of affected skin, revealing the known somatic pathogenic variant in *HRAS* (NM_005343.4(*HRAS*):c.37G > C (p.Gly13Arg)). Symptomatic treatment, including physical therapy, occupational therapy and dysphagia therapy, was initiated.

At 6 months of age, corrective eye muscle surgery was performed to address bilateral strabismus. By 10 months, the infant developed redness, tortuosity of the episcleral vessels and protrusio bulbi of the left eye, leading to the diagnosis of orbital embryonal rhabdomyosarcoma (IRS III) without metastases. She was treated with standard chemotherapy according to the CWS Guidance (SRG, Subgroup C)[22]. Nevertheless, after a good initial response, she showed a rapid progression of her tumor at the end of the intense chemotherapy. Radiation therapy with protons (50.4 Gy) was administered, followed by second-line chemotherapy (ACCTTIVE) according to CWS Guidance[23]. Unfortunately, a new rapid tumor progression despite therapy indicated radical surgery with orbital exenteration and adjuvant Carbon Ion Therapy (45 Gy) as individual curative attempt. The oncological disease is in complete remission 15 months after the end of therapy.

**Fig. 2:**
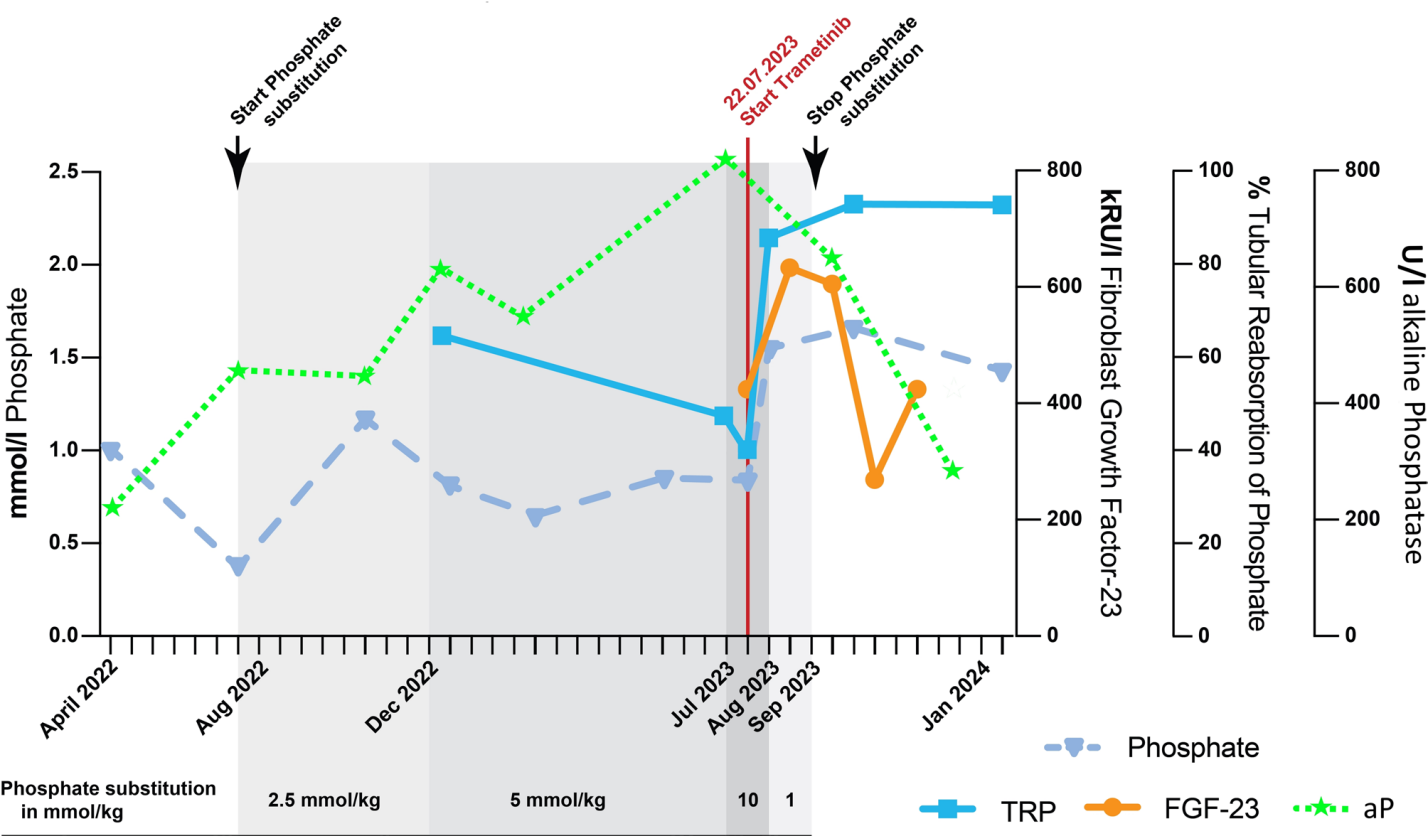
Management of hypophosphatemia. Grey background shows dosage of phosphate substitution, which was started as orally, divided into four equal dosages. Increasing phosphate losses eventually demanded hospitalization with intravenous continuous phosphate substitution

**Fig. 3:**
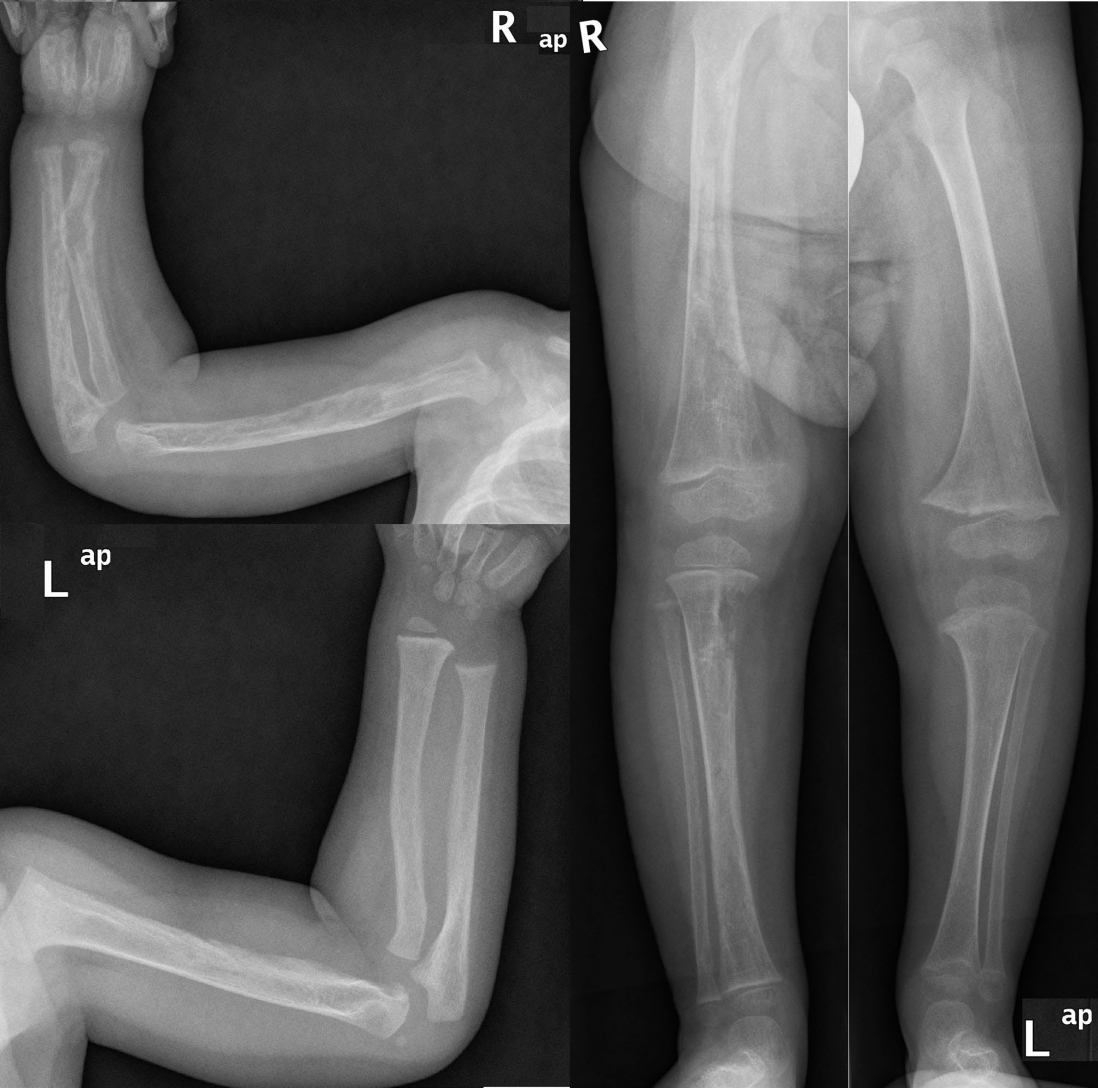
Asymmetric osteomalacia predominantly affecting the right-sided extremities

During the initial stages of the first-line therapy, a significant hypophosphatemia of 0.37 mmol/L (Ref 1.0–1.95) was identified through routine assessments at the age of 13 months (Fig. 2). Alkaline phosphatase (AP) was elevated at 442 U/L (Ref 96–311), while calcium levels were within the normal range. Consequently, an oral substitution was initiated with phosphate at 2.5 mmol/kg/day divided into four doses. At that time, the child received a prophylactic dose of 500 IU/day of Vitamin D3. Initially, the phosphate serum levels stabilized within the lower normal range for six months. At the age of 18 months the phosphate serum level subsequently decreased to 0.81 mmol/L. At this point, tubular phosphate reabsorption (TRP) was reduced to 61% (Ref 85–99%), parathyroid hormone (PTH) was elevated to 12.4 pmol/L (Ref 1.3–7.6), 25 OH-Vitamin D3 (Calcidiol) was 49.1 ng/mL (Ref 6.3–46.4), and AP increased to 627 U/L (Ref 96–311). In response, the substitution of Vitamin D3 was increased to 1000 IU/day, and additional doses of Alfacalcidol at 0.025 µg/kg/day were started. The oral phosphate substitution was consistently adjusted to 5 mmol/kg/day divided into four doses (Fig. 2). This adjustment helped to maintain the phosphate levels within the lower end of the normal range until the age of 24 months. Then, reevaluation of the calcium and phosphate balance showed PTH at 42 pmol/L (Ref 1.3–7.6), AP at 635 U/L (Ref 96–311), TRP at 46.83% (Ref 85–99), and normal calcium levels, prompting further increase of Alfacalcidol to 0.05 µg/kg/day. At the age of 25 months, an X-ray of the right arm was conducted due to palpable swelling during the clinical examination, revealing significant bone structure abnormalities with osteopenia and several pathological fractures of varying ages. Expanded X-ray screening revealed predominant rickets in the right extremities, right-sided thoracic bones, and right os pubis, with only minimal changes in the left arm and leg (Fig. 3). Fibroblast growth factor 23 (c-terminal) (FGF23) was subsequently measured at 424 kRU/L (Ref 26–110) and AP reached the maximum at 812 U/L (Ref 96–311), TRP decreased to 40% (Ref 85–99%) (Fig. 2), which led to another doubling of the Alfacalcidol dose (0.1 µg/kg/day). At this point, 13 months after starting the oral supplementation, she required a phosphate substitution of 10 mmol/kg/day, which was given continuously intravenously due to gastrointestinal side effects.

As RAS activation is known to induce FGF23 and thus hypophosphatemia through diminishing phosphate reabsorption and increasing urinary phosphate excretion, treatment with the MEK inhibitor trametinib was started at a dosage of 0.025 mg/kg/day (0,25mg) in a hospital setting at the age of 26 months [24]. Informed consent for off-label use of trametinib was obtained from parents after detailed explanation of potential risks, benefits, and unknowns associated with the off-label. Treatment was approved by a multidisciplinary hospital board, including neuropediatricians, oncologists, and endocrinologists. For dosing and safety assessment in childhood, our pharmacological board reviewed treatment and follow-up safety protocols.

**Fig. 4:**
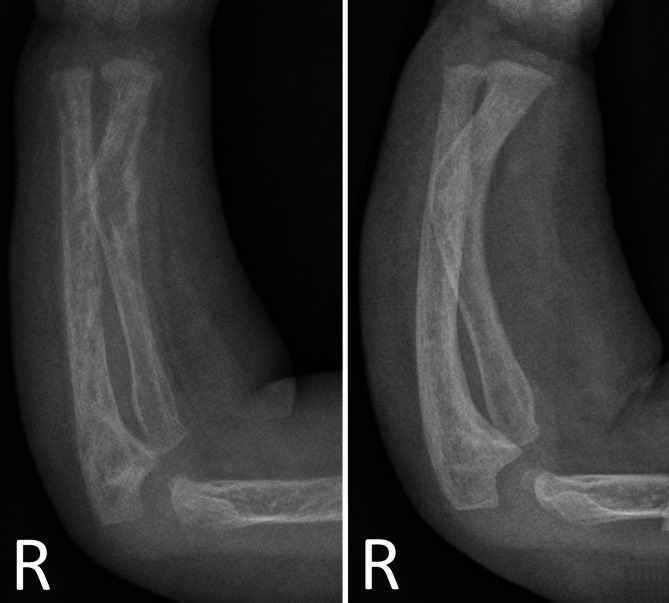
X-ray follow-up of right upper extremity, before and after trametinib therapy

Within a week the intravenous phosphate substitution could be stopped and decreased to 1 mmol/kg/day, given orally divided into four doses. The TRP increased from 40 to 80% (Ref 85–99), the PTH decreased from 46 pmol/L to 17 pmol/L (Ref 1.3–7.6), and alfacalcidol was switched to calcitriol at 50% of the previous dose (0.05 µg/kg/day). After one month of treatment with trametinib, the phosphate substitution was stopped. The current medication comprises calcitriol and trametinib. Under this therapy, the phosphate levels are normal, with a TRP of 92.76% (range 85–99). AP consistently decreased after the initiation of therapy with trametinib and normalized five months after the start of treatment at the age of 30 months (from 812 U/L to 280 U/L (Ref 96–311)) (Fig. 2). On the X-ray two month after start of trametinib rickets improved significantly (Fig. 4).

Prior to therapy with trametinib, she could only sit and showed significant muscular hypotonia. During the first three months of treatment with the MEK inhibitor, the child experienced rapid developmental progress, now being able to walk freely, showing significant motor activity, good coordination and normal cognition.

## Discussion

In this case report, we present the first case of a child with HRAS-related mosaic RASopathy presenting as SFMS and associated severe hypophosphatemia who was successfully treated with trametinib. MEK-inhibitor treatment is a promising targeted therapy for RASopathies with the potential of disease modification. It has been used in Noonan syndrome, Neurofibromatosis type 1, various oncological diseases and manifestations like cardiomyopathy and epilepsy[25, 26, 27].

At 25 months of age rickets with rapidly progressive skeletal demineralization, pathological fractures, muscle weakness and severe hypophosphatemia became apparent in our girl. As no response to phosphate and vitamin D supplementation was observed, despite sustained oral and intravenous high doses to compensate for the high renal loss, alternative treatment options were considered. Treatment with the anti-FGF23 antibody burosumab, approved for treating X-linked hypophosphatemia[28], has alternatively been described in HRAS-associated hypophosphatemia[29]. Considering multisystem manifestations of the underlying RASopathy in our child, including aggressive orbital rhabdomyosarcoma that was resistant to radiochemotherapy and required radical mutilating surgery, we chose MEK-inhibitor treatment with trametinib to modulate the general molecular pathogenetic mechanism from the HRAS genetic mosaicism.

Considering the causes of hypophosphatemia, numerous reports, both in vitro and in vivo, have demonstrated RAS activation-induced FGF23 elevation [17, 30, 31]. For a detailed review on the possible molecular pathomechanisms we refer to Takashi et al. (2019)[32]. As FGF23 is known to cause hypophosphatemia through the downregulation of NPT2a and NPT2c in the proximal renal tubules, the marked hypophosphatemia in this child was likely caused by RAS activation. This mechanism and the somatic *HRAS* gain-of-function variant also explain the asymmetric and regional occurrence of rickets. In contrast, tumor-induced osteomalacia is a rare paraneoplastic syndrome caused by tumoral production FGF23. If the tumor cannot be resected, novel therapeutic approaches include treatment with the anti-FGF23 antibody burosumab [29].

Given the good tolerance of MEK inhibitory treatment, therapy with trametinib is ongoing, and the girl is constantly showing developmental progress and no signs of cancer relapse 29 months after the initial diagnosis of embryonal rhabdomyosarcoma. In addition, she showed improvements in texture and color of skin manifestations and improved growth of hair on affected skin. The severe rickets improved rapidly after two months of treatment. We monitor trametinib therapy as recommended within a multidisciplinary framework [27]. In addition to clinical evaluations and laboratory assessments, regular ophthalmologic and pediatric cardiology consultations are conducted. Oncological follow-up is performed according to the recommendations of the CWS study group[22]. All assessments to date were unremarkable. Since there are currently no recommendations for therapy discontinuation, treatment should continue under active monitoring, considering the risk-benefit profile in the presence of multisystemic manifestations, including oncological components. The decision regarding relevant dose adjustment or possible discontinuation of trametinib should be made in a multidisciplinary manner[27].

Our case highlights the use of MEK inhibitor therapy in mosaic RASopathies as an additional targeted treatment option.

## Data Availability

All relevant data and material have been included in this report.

## Abbreviations

SFMS: Schimmelpenning-Feuerstein-Mims syndrome
MAPK: Mitogen-Activated Protein Kinase
eRMS: Embryonal Rhabdomyosarcoma
CSHS: Cutaneous skeletal hypophosphatemia
TRP: Tubular Phosphate Reabsorption
